# Alcohol consumption and cigarette smoking in relation to high frequency of p53 protein accumulation in oesophageal squamous cell carcinoma in the Japanese

**DOI:** 10.1054/bjoc.1999.1212

**Published:** 2000-06

**Authors:** H Saeki, S Ohno, K Araki, A Egashira, H Kawaguchi, Y Ikeda, M Morita, K Kitamura, K Sugimachi

**Affiliations:** The Second Department of Surgery, Faculty of Medicine, Kyushu University, 3-1-1 Maidashi, Higashi-ku, Fukuoka, 812-8582, Japan

**Keywords:** alcohol, cigarettes, p53, oesophageal squamous cell carcinoma

## Abstract

We investigated levels of p53 protein expression in Japanese patients with oesophageal squamous cell carcinoma. A significantly larger proportion of heavy alcohol drinkers and cigarette smokers was evident in the p53-positive group. The combination of drinking and smoking was associated with a high frequency of p53 protein accumulation. © 2000 Cancer Research Campaign


*British Journal of Cancer* (2000) 82(**11**), 1892–1894
© 2000 Cancer Research Campaign
DOI: 10.1054/ bjoc.1999.1212, available online at http://www.idealibrary.com on IDEAL®

### Alcohol consumption and cigarette smoking in relation to high frequency of p53 protein accumulation in oesophageal squamous cell carcinoma in the Japanese

**H Saeki, S Ohno, K Araki, A Egashira, H Kawaguchi, Y Ikeda, M Morita, K Kitamura and K Sugimachi**

The Second Department of Surgery, Faculty of Medicine, Kyushu University, 3-1-1 Maidashi, Higashi-ku, Fukuoka 812-8582, Japan

**Summary** We investigated levels of p53 protein expression in Japanese patients with oesophageal squamous cell carcinoma. A significantly larger proportion of heavy alcohol drinkers and cigarette smokers was evident in the p53-positive group. The combination of drinking and smoking was associated with a high frequency of p53 protein accumulation. © 2000 Cancer Research Campaign

**Keywords**: alcohol; cigarettes; p53; oesophageal squamous cell carcinoma

Alcohol drinking and cigarette smoking are considered to be important risk factors of oesophageal squamous cell carcinoma in the Japanese. Alcohol consumption was associated with an increased risk of oesophageal cancer, the amount consumed being the main risk determinant (Hanaoka et al, 1994). A statistically significant dose–response relationship between cigarette smoking and oesophageal cancer in men has been reported (Akiba and Hirayama, 1990).

In oesophageal squamous cell carcinomas, loss of 17p and mutations of the p53 gene are frequent, being detected in approximately one-half the cases studied (Wagata et al, 1993). Immunohistochemical analyses using specific antibodies to p53 showed that the abnormal accumulation of p53 protein was evident at pre-cancerous stages, suggesting their importance even in the early stage of carcinogenesis (Bennett et al, 1992; Wang et al, 1993; Gao et al, 1994).

Expression of p53 protein in oesophageal squamous cell carcinoma tissues taken from Japanese patients was investigated immunohistochemically and the relation of p53 protein accumulation to the patient's history of alcohol consumption and cigarette smoking was analysed.

### MATERIALS AND METHODS

Our study was based on 126 consecutive individuals with primary oesophageal squamous cell carcinoma surgically treated in the Second Department of Surgery, Kyushu University in Japan. None of these patients had been given preoperative irradiation or chemotherapy. To define the use of alcohol and cigarettes, we used the same questionnaire for all patients, as described previously (Morita et al, 1994). One drink of alcohol corresponded to 180 ml of sake (the most popular rice wine alcoholic beverage in Japan), 120 ml of white liquor (shochu), 70 ml of whisky and 720 ml of beer. A 'drinking index' of accumulated amount of ethanol was defined as number of drinks per week × number of years of drinking. 'Pack-years' of smoking was defined as (number of cigarettes per day/20) × number of years of smoking.

p53 immunohistochemical staining was performed on formalin-fixed, paraffin-embedded oesophageal tissue from all patients. The sections were dewaxed in xylene and rehydrated with ethanol and then microwaved at 93°C in phosphate-buffered saline. These sections were incubated with 1% hydrogen peroxide in methanol to block endogenous peroxidase activity. Normal rabbit serum was applied for 20 min to reduce any non-specific antibody binding. A mouse monoclonal antibody against the p53 (DO-7, Dako, Glostrup, Denmark) with a 1:100 dilution was incubated overnight at 4°C. Staining was detected using the streptavidin–biotin–peroxidase complex method (Histofine SAB kit, Nichirei, Tokyo, Japan). p53 protein accumulation was investigated in each specimen by investigators blind to the risk factor data. When the immunoreactive neoplastic cells accounted for over 10%, a positive result was recorded.

Data on patient groups were compared using either $\chi^2$ test or Student's *t*-test. To evaluate the correlation between risk factor data and p53 protein accumulation, the odds ratios (ORs) for alcohol and smoking variables as well as their 95% confidence intervals (CIs) were calculated. A *P*-value of < 0.05 was considered significant.

### RESULTS

Among 126 patients with oesophageal squamous cell carcinoma, 70 (55.6%) belonged to the p53 protein accumulation positive group. There were no significant differences between the p53 protein accumulation positive and negative groups with regard to age, sex, location of the main tumour, differentiation and TNM stage.

Table 1 shows the correlation between the history of alcohol consumption, cigarette smoking and p53 protein accumulation. In the p53 protein accumulation positive group, the number of heavy drinkers was significantly larger than in the negative group. Relative to non-drinkers the OR for those who consumed 20 or more drinks per week was 4.8, which increased to 5.9 for those with a drinking index of 700 or more. With respect to the type of

*Received 5 October 1999*
*Revised 29 November 1999*
*Accepted 30 November 1999*

*Correspondence to:* H Saeki

**Table 1** Correlation between the history of alcohol consumption, cigarette smoking and p53 protein accumulation

| | p53 protein accumulation | | OR | 95% CI |
|---|---|---|---|---|
| | Negative ($n$ = 56) | Positive ($n$ = 70) | | |
| Number of drinks per week | | | | |
| 0 | 11 | 6 | 1.0 | – |
| >0, < 20 | 32 | 30 | 1.7 | 0.6–5.2 |
| ≥ 20 | 13 | 34 | 4.8 | 1.5–14.9 |
| Most often consumed drink | | | | |
| Beer | 11 | 10 | 1.7 | 0.5–6.2 |
| Sake (rice wine) | 21 | 35 | 3.1 | 1.0–9.2 |
| Whisky | 5 | 8 | 2.9 | 0.7–12.8 |
| White liquor (shochu) | 8 | 11 | 2.5 | 0.7–9.6 |
| Drinking index[a] | | | | |
| ND: 0 | 11 | 6 | 1.0 | – |
| LD: >0, <700 | 35 | 32 | 1.7 | 0.6–5.0 |
| HD: > = 700 | 10 | 32 | 5.9 | 1.8–18.4 |
| Cigarettes per day | | | | |
| 0 | 19 | 15 | 1.0 | – |
| >0, < 25 | 24 | 27 | 1.4 | 0.6–3.4 |
| ≥25 | 13 | 28 | 2.7 | 1.1–6.9 |
| Pack-years[b] | | | | |
| NS: 0 | 19 | 15 | 1.0 | – |
| LS: >0, <50 | 28 | 28 | 1.3 | 0.5–3.0 |
| HS: ⩾ 50 | 9 | 27 | 3.8 | 1.4–10.3 |
| Drinking index[a]/pack-years[b] | | | | |
| ND/NS | 7 | 4 | 1.0 | – |
| LD/NS | 10 | 6 | 1.1 | 0.2–4.8 |
| HD/NS | 2 | 5 | 4.4 | 0.6–32.1 |
| ND/LS | 3 | 1 | 0.6 | 0.0–7.6 |
| LD/LS | 18 | 17 | 1.7 | 0.4–6.6 |
| HD/LS | 7 | 10 | 2.5 | 0.5–11.7 |
| ND/HS | 1 | 1 | 1.8 | 0.1–35.4 |
| LD/HS | 7 | 9 | 2.3 | 0.5–10.7 |
| HD/HS | 1 | 17 | 29.8 | 4.2–210.9 |

[a]Number of drinks per week × number of years of drinking; ND: non-drinkers, LD: light drinkers, HD: heavy drinkers. [b](Number of cigarettes per day/20) × number of years of smoking; NS: non-smokers, LS: light smokers, HS: heavy smokers.

drink, the OR for those who usually consumed sake increased to 3.1. In the p53 protein accumulation positive group, there was a significantly larger proportion of heavy smokers than in the negative group. The OR for those who smoked 25 or more cigarettes per day was 2.7, which increased to 3.8 for those who had smoked 50 or more cigarettes per day × year (pack-years). The combination of use of alcohol and cigarettes was clearly associated with a high frequency of the p53 protein accumulation. p53 protein accumulation was noted in 17 of 18 in patients with a history of heavy smoking and drinking, the OR being 29.8.

### DISCUSSION

Our findings show that accumulation of p53 protein is closely associated with both large amounts of alcohol and cigarette use, to our knowledge the first such report. The carcinogens in alcohol and cigarettes affects to mutate p53 in such a way as to increase protein accumulation, and there may be both a loss of suppressor gene function and a gain in the dominant transforming activity of p53. Cigarette smoke is a complex mixture of thousands of carcinogens (DeMarini, 1983) and the association between cigarette consumption and mutation of p53 in cancer has been noted previously (Suzuki et al, 1992; Brennan et al, 1995).

Our findings show a clear association between alcohol drinking and p53 protein accumulation and we suggest an interrelationship between alcohol and cigarette use. It seems likely that carcinogens included in cigarette smoke remain in the oral cavity and are taken into the oesophagus with alcohol drinking by heavy smokers and drinkers. Over 30 years ago, it was reported that benzo(a)pyrene dissolves in aqueous solutions of ethanol and can enter the epithelium of the oesophagus, as evidenced in an animal model (Kuratsune et al, 1965); this may explain the interrelationship between alcohol and cigarette use in our patients. However, to more accurately evaluate the respective roles and the combined effects of these factors, a larger study using a multivariate analysis would be needed.

Immunohistochemical screening for elevated protein levels followed by sequence analysis represents an efficient strategy for elevation of the p53 mutational spectrum (Bennett et al, 1991; Bodner et al, 1992). Sequence analysis should further elucidate the relation between risk factors and mutation of p53 in oesophageal squamous cell carcinoma.

Our findings show that alcohol and cigarette use is related to high frequency of p53 protein accumulation in oesophageal squamous cell carcinoma in the Japanese. Our results suggest the aetiological contribution of exogenous factors to oesophageal carcinogenesis may have implications for cancer risk assessment. We conclude that the p53 tumour suppressor gene is the candidate

*© 2000 Cancer Research Campaign*

molecular target of exposure to alcohol and cigarettes in oesophageal carcinogenesis.

### ACKNOWLEDGEMENTS

We are grateful to K Akazawa for assistance with the statistical analyses. This study was supported by grants from the Ministry of Education, Science, Sports and Culture of Japan.

### REFERENCES

Akiba S and Hirayama T (1990) Cigarette smoking and cancer mortality risk in Japanese men and women – results from reanalysis of the six-prefecture cohort study data. *Environ Health Perspect* **87**: 19–26

Bennett WP, Hollstein MC, He A, Zhu SM, Resau JH, Trump BF, Metcalf RA, Welsh JA, Midgley C, Lane DP and Harris CC (1991) Archival analysis of p53 genetic and protein alterations in Chinese esophageal cancer. *Oncogene* **6**: 1779–1784

Bennett WP, Hollstein MC, Metcalf RA, Welsh JA, He A, Zhu SM, Kusters I, Resau JH, Trump BF, Lane DP and Harris CC (1992) p53 mutation and protein accumulation during multistage human esophageal carcinogenesis. *Cancer Res* **52**: 6092–6097

Bodner SM, Minna JD, Jensen SM, D'Amico D, Carbone D, Mitsudomi T, Fedorko J, Buchhagen DL, Nau MM, Gazdar AF and Linnoila RI (1992) Expression of mutant p53 proteins in lung cancer correlates with the class of p53 gene mutation. *Oncogene* **7**: 743–749

Brennan JA, Boyle JO, Koch WM, Goodman SN, Hruban RH, Eby YJ, Couch MJ, Forastiere AA and Sidransky D (1995) Association between cigarette smoking and mutation of the p53 gene in squamous-cell carcinoma of the head and neck. *N Engl J Med* **332**: 712–717

DeMarini DM (1983) Genotoxicity of tobacco smoke and tobacco smoke condensate. *Mutat Res* **114**: 59–89

Gao H, Wang LD, Zhou Q, Hong JY, Huang TY and Yang CS (1994) p53 tumor suppressor gene mutation in early esophageal precancerous lesions and carcinoma among high-risk populations in Henan, China. *Cancer Res* **54**: 4342–4346

Hanaoka T, Tsugane S, Ando N, Ishida K, Kakegawa T, Isono K, Takiyama W, Takagi I, Ide H, Watanabe H and et al. (1994) Alcohol consumption and risk of esophageal cancer in Japan: a case–control study in seven hospitals. *Jpn J Clin Oncol* **24**: 241–246

Kuratsune M, Kohchi S and Horie A (1965) Carcinogenesis in the esophagus I. Penetration of benzo[a]pyrene and other hydrocarbons into the esophageal mucosa. *Gann* **56**: 177–187

Morita M, Kuwano H, Ohno S, Sugimachi K, Seo Y, Tomoda H, Furusawa M and Nakashima T (1994) Multiple occurrence of carcinoma in the upper aerodigestive tract associated with esophageal cancer: reference to smoking, drinking and family history. *Int J Cancer* **58**: 207–210

Suzuki H, Takahashi T, Kuroishi T, Suyama M, Ariyoshi Y, Takahashi T and Ueda R (1992) p53 mutations in non-small cell lung cancer in Japan: association between mutations and smoking. *Cancer Res* **52**: 734–736

Wagata T, Shibagaki I, Imamura M, Shimada Y, Toguchida J, Yandell DW, Ikenaga M, Tobe T and Ishizaki K (1993) Loss of 17p, mutation of the p53 gene, and overexpression of p53 protein in esophageal squamous cell carcinomas. *Cancer Res* **53**: 846–850

Wang LD, Hong JY, Qiu SL, Gao H and Yang CS (1993) Accumulation of p53 protein in human esophageal precancerous lesions: a possible early biomarker for carcinogenesis. *Cancer Res* **53**: 1783–1787

*© 2000 Cancer Research Campaign*
